# Users taking the blame? How service failure, recovery, and robot design affect user attributions and retention

**DOI:** 10.1007/s12525-022-00613-4

**Published:** 2023-01-19

**Authors:** Nika Meyer (née Mozafari), Melanie Schwede, Maik Hammerschmidt, Welf Hermann Weiger

**Affiliations:** 1 University of Goettingen, Smart Retail Group, Platz Der Goettinger Sieben 3, 37073 Goettingen, Germany; 2 Alfaisal University, Riyadh, Kingdom of Saudi Arabia

**Keywords:** Human–robot interaction, Service failure, Service recovery, Social cognition, Responsibility attribution, User retention, M31, O32, I10

## Abstract

Firms use robots to deliver an ever-expanding range of services. However, as service failures are common, service recovery actions are necessary to prevent user churn. This research further suggests that firms need to know how to design service robots that avoid alienating users in case of service failures. Robust evidence across two experiments demonstrates that users attribute successful service outcomes internally, while robot-induced service failures are blamed on the firm (and not the robot), confirming the well-known self-serving bias. While this external attributional shift occurs regardless of the robot design (i.e., it is the same for warm vs. competent robots), the findings imply that service recovery minimizes the undesirable external shift and that this effect is particularly pronounced for warm robots. For practitioners, this implies prioritizing service robots with a warm design for maximizing user retention for either type of service outcome (i.e., success, failure, and failure with recovery). For theory, this work demonstrates that attribution represents a meaningful mechanism to explain the proposed relationships.

**Supplementary Information:**

The online version contains supplementary material available at 10.1007/s12525-022-00613-4.

## Introduction

Over the past couple of years, service robots have been vigorously changing the service landscape (De Keyser & Kunz, 2022). Service robots are “system-based autonomous and adaptable interfaces that interact, communicate and deliver service to an organization's customers” (Wirtz et al., 2018, p. 909). Applications range from retail (Meyer et al., 2020), hotels and restaurants (Belanche et al., 2020; Choi et al., 2020) to hospitality and healthcare services (Čaić et al., 2018; Yoganathan et al., 2021). Especially the latter have gained importance as the Covid-19 pandemic transforms the digital economy, where service delivery becomes increasingly technology-mediated (Ågerfalk et al., 2020; S. Kim et al., 2021; Koo et al., 2021). In fact, the market for professional service robots has grown worldwide by 12% in 2020 (International Federation of Robotics, 2021). Initial research efforts mirror this practical relevance and investigate the design of service robots (e.g., Zhang et al., 2022; K. J. Kim et al., 2013) and users’ reactions to them (e.g., Belanche et al., 2021; Choi et al., 2020). In fact, reviews of extant literature in the field call for more research that investigates human–robot interactions (HRI), for example, in terms of user responses to robo-advisors (De Keyser & Kunz, 2022; Lim et al., 2022).

Service robots can take on tasks of high cognitive-analytical complexity (Wirtz et al., 2018) and are currently taking great leaps toward performing tasks that require high empathetic intelligence (Huang & Rust, 2018). However, while firms invest in creating effective HRI, reality proves that service robots are often prone to failures, which risks adverse robot- and firm-related outcomes (Honig & Oron-Gilad, 2018). We define service failures as situations in which the service delivery by a robot does not result in the desired service outcome (Smith et al., 1999). To better understand how users react to robot-induced failures, our research investigates how users attribute responsibility for such failures (compared to successful service outcomes). Based on attribution theory, we assume that responsibility attribution is an important explanatory mechanism that indicates how users evaluate service outcomes and how this influences their behavior. Previous research indicates that internal responsibility attribution can positively affect users' perceptions and behavior toward service agents and firms (e.g., Leung et al., 2018; Yalcin et al., 2022). Therefore, firms need to understand how robots can facilitate this internal responsibility attributions if they act as service agents. Alarmingly, in the context of service robots, an examination of internal responsibility attribution (i.e., whether users think they are responsible for creating the service outcome) is still missing. Previous research in the service robot field focused predominantly on external responsibility attribution regarding service robots and firms (Belanche et al., 2020; Leo & Huh, 2020) and showed controversial results in relation to the self-serving bias (Jörling et al., 2019). This discrepancy may be a result of inconsistent conceptualizations of “externality”, as it can refer to a broad variety of external actors. However, with internal responsibility attribution, the object of reference is unambiguous, while it still represents a theoretical counterpart to external responsibility attribution. Therefore, internal responsibility attribution should be a helpful explaining mechanism to shed light onto these prior adverse findings.

Furthermore, service research has long recognized that fully eliminating failures from service interactions is “an insurmountable task” (Webster & Sundaram, 1998, p. 153) because the technology today is at a level where it is prone to failure (Honig & Oron-Gilad, 2018). Therefore, it is crucial to find ways of effectively recovering from robot-induced service failures (Choi et al., 2020), and to our knowledge, there is no prior research on the effects of recovery on responsibility attribution and subsequent user responses in HRI.

Moreover, previous HRI research has shown that different robot types can lead to different user responses (Belanche et al., 2021). More specifically, the two central dimensions of human social cognition—warmth and competence (Fiske et al., 2007)—promise to be effective in explaining user reactions to service robots (van Doorn et al., 2017). Therefore, this work examines the effect of service robot design with a focus on warmth vs. competence perceptions. Further, we consider whether the effects of warm vs. competent service robot design on responsibility attributions vary depending on different service outcomes, as this remains largely unexamined in extant research. Notably, prior work investigating user attributions in interactions with service robots has predominantly focused on comparing failures committed by service robots to failures committed by humans (e.g., Belanche et al., 2020; Merkle, 2019). However, human-like features of service robots could change responsibility attribution after different service outcomes, which merits further research (Leo & Huh, 2020; Yam et al., 2021). Therefore, we examine how warm and competent robot design affects user attributions after service failure and recovery.

Finally, prior studies investigating the repercussions of different service outcomes for user attributions have stopped at this stage of the effect chain, and how these attributions affect subsequent user behavior has been addressed only to a limited extent (Jörling et al., 2019). However, insights on behavioral outcomes are needed to offer firms actionable implications. Puntoni et al. (2021) note that adverse service outcomes in interactions with AI-based technologies will alienate users to the point that it harms the user-firm relationship. Therefore, user retention represents a relevant behavioral outcome that merits further research attention as it has crucial profitability relevance for firms. Interactions with service robots typically involve the user, the firm, and the service robot as the three key actors (Wirtz et al., 2018). From a service provider's perspective, user retention can accordingly be recognized on a robot-level (i.e., the user's intention to reuse the service robot) and on a firm-level (i.e., the user's intention to stay loyal toward the firm) (Palmatier et al., 2007). Prior work has demonstrated that disentangling these actors provides a more holistic view on the repercussions of different service outcomes in interactions with service robots (Belanche et al., 2021; Leo & Huh, 2020).

Hence, this study's overarching research goal is to examine the effects of different service outcomes (i.e., success, failure, failure with recovery) and different service robot designs (i.e., warm vs. competent service robot design) on internal responsibility attribution and subsequent robot-level and firm-level user retention. To achieve this goal, we conduct two studies that contribute to research on service delivery with robots in several ways. First, we contribute to research on attribution theory by providing evidence that triggering internal responsibility attribution has a positive impact on robot-level and firm-level user retention. Second, while our studies confirm the existence of a self-serving bias in a service robot context (i.e., claiming success to oneself, but blaming external circumstances for failures), the results are unique in showing that although failures are committed by robots, blame for the failure is shifted to the firm. Third, we demonstrate that service recovery through human handover mitigates the deleterious externalization of attribution by shifting back attribution internally and getting users to take their share of responsibility for failures that are created during their interactions with robots. Fourth, by applying concepts from social cognition theory, we demonstrate that robot design affects attributional thinking. Specifically, users internalize responsibility for an outcome if the service robot has warm (vs. competent) design features. While users are indifferent to the type of robot in case of a non-resolved failure, service robot design alters how users attribute responsibility for a recovered failure in that they are more forgiving toward robots with warm design. Together, these findings indicate that deploying warm robots should be the dominant strategy for service providers as they are – across all possible service outcomes – either superior or equally effective as competent robots. These findings are robust across two experiments in two different medical contexts, one context involves the communication of a medical diagnosis and the other a medical product recommendation by the service robot. Thus, our study contributes to traditional HRI research as well as to recent HRI research on recommendations by robo-adivsors.

## Theoretical background

**Fig. 1:**
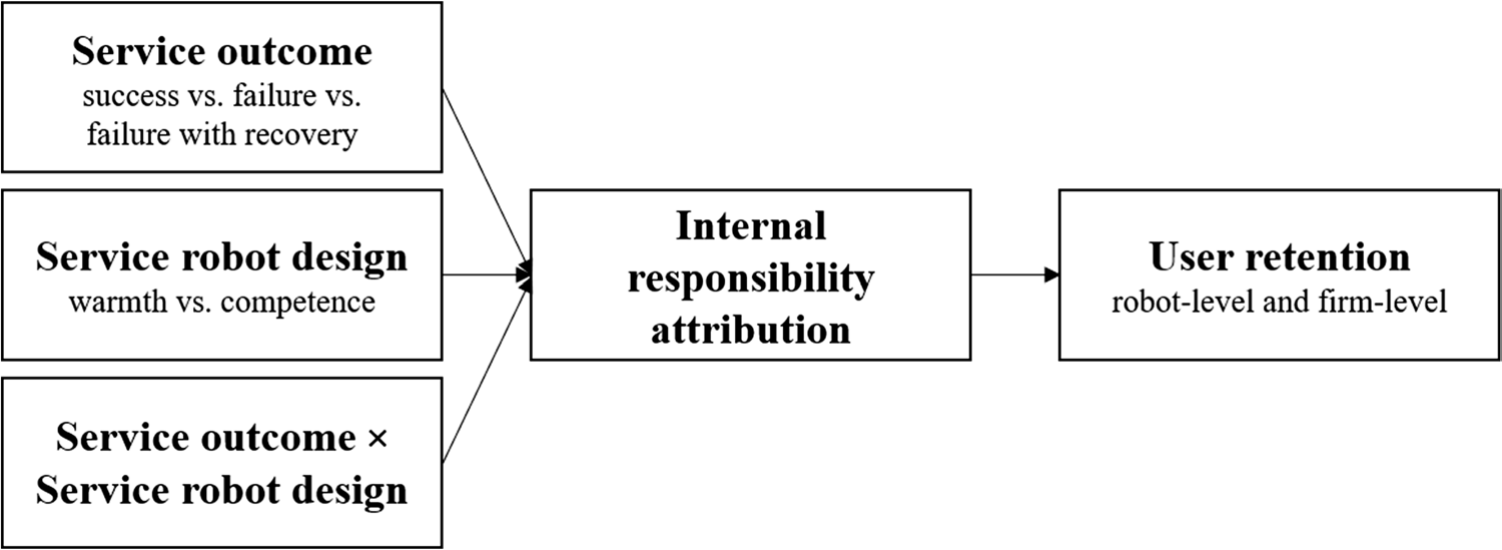
Research framework

The following section provides the theoretical background for our research and hypothesis development. First, we present attribution theory, derive the relevance of internal responsibility attribution for user retention, and elaborate on how different service outcomes influence internal responsibility attribution. Second, we use social cognition theory to delineate different types of service robots (warm vs. competent) and illustrate the effect of service robot design on internal responsibility attribution. Finally, we discuss the interaction of service outcome and service robot design. Figure 1 depicts our research framework.

### Attribution theory

To understand how different service outcomes affect user retention, we rely on attribution theory. The following section discusses the relevance of internal responsibility attribution for user retention and presents how it is triggered by successful service provision, service failure, and service recovery. Based on theoretical considerations and prior empirical work, we derive our hypotheses for the effect of internal responsibility attribution on user retention at the robot- and firm-level, as well as different service outcomes on internal responsibility attribution.

#### The relevance of internal responsibility attribution for user retention

Attribution theory considers “how people arrive at causal inferences, what sort of inferences they make, and what the consequences of these inferences are” (Folkes, 1988, p. 548). Therefore, attribution theory allows us to analyze how users assign blame when a service failure happens and if they claim responsibility for service outcomes to themselves (Harris et al., 2006). In attribution theory, this refers to the term responsibility attribution, which describes who or what caused the service outcome and whether this entity could have had control over the outcome or not (Belanche et al., 2020; Folkes, 1988; Hamilton, 1978). Users can attribute an outcome, whether failure or success, internally, to themselves, or externally, to the firm, service robot, employee, or other causes (Belanche et al., 2020; Lei & Rau, 2021; Weiner, 2000).

Service robot outcomes will significantly determine user retention (Mozafari et al., 2022). We assume that internal responsibility attribution is an important mechanism to explain user retention on the robot-level and firm-level in response to different service outcomes. Robot-level retention is an important short-term outcome as it enhances acceptance of technology in replacing human employees for service delivery (Wirtz et al., 2018), and firm-level retention is particularly important due to positive long-term performance effects for firms (Rajaobelina et al., 2021). Moreover, studies have already shown that internal responsibility attribution influences users' behavior (e.g., Leung et al., 2018), users' perceptions of the firm's performance (e.g., Dunn & Dahl, 2012), users' attitude toward the firm (Yalcin et al., 2022), and satisfaction with the technology (Heidenreich et al., 2015).

If users attribute the responsibility of a service outcome to themselves, they feel like they are in control and have contributed to the service outcome (van Raaij & Pruyn, 1998). Personal control, the feeling that outcomes are determined by oneself and not merely by others (Puntoni et al., 2021), is a basic need and a precondition for well-being (Leotti et al., 2010). Therefore, internal responsibility attribution empowers users in a service interaction. Increased control leads users to experience positive emotions (Shapiro et al., 1996), which makes them more willing to use the technology as well as more satisfied with the service experience (Jörling et al., 2019; van Raaij & Pruyn, 1998). Instead, loss of control results in reactance toward the technology and firm (Puntoni et al., 2021). To sum it up, internal attributions of service outcomes determine the perception of service quality and reactions such as (dis)satisfaction (van Raaij & Pruyn, 1998), which are central for user retention (Anderson & Mittal, 2000).

Accordingly, we formulate the following hypothesis:

- H_1_: Internal responsibility attribution increases (a) robot-level and (b) firm-level user retention.

#### Service failure and responsibility attribution

As noted above, users attribute responsibility to make sense of different outcomes. A tenet of attribution theory is that attributions are prone to biases – the most prominent bias is the self-serving bias. This bias describes the tendency of users to explain negative outcomes externally and positive outcomes internally. In attributing successful outcomes to oneself and blaming others for failures, individuals strive to perpetuate their self-worth (Miller & Ross, 1975). Studies show that this bias does not only apply in relation to social interactions with humans but also in technology-based interactions (Lei & Rau, 2021; Moon, 2003; Yalcin et al., 2022). Users attribute successful outcomes to themselves because of their unique skills and characteristics, which humans, not technologies, possess. Moreover, users do not think that technologies can fulfill their individual needs to a satisfactory degree because, for technology, every user is just a number (Yalcin et al., 2022). This is also reflected in failure outcomes, as users can easily blame technologies for failing to meet individual user needs (Longoni et al., 2019), so they are less likely to attribute undesirable outcomes to themselves.

In the context of service robots specifically, a limited number of studies has investigated the effect of service failure on responsibility attribution. For example, Leo and Huh (2020) find that users attribute responsibility for service robot failures more to the firm and less to the robot, while the opposite is the case if the failure was committed by a human employee. These findings are corroborated by Belanche et al. (2020), who further show that service robot failures are perceived as more stable than human failures, suggesting that users identify the robot's programming by the firm as the cause for failure. However, Jörling et al. (2019) demonstrate the opposite of the self-serving bias: positive outcomes in interactions with service robots are attributed more externally and negative outcomes more internally. These findings indicate that more research is necessary to prove when the self-serving bias emerges in the service robot context and when not.

Notably, prior work focuses on the emergence of external forms of responsibility attribution, neglecting how and when users assign responsibility to themselves. Specifically, the effect of service failures on internal responsibility attribution remains unexplored in a service robot context. However, as established above, internal responsibility attribution is central to achieving desirable robot-related and firm-related outcomes. Since previous studies in other contexts suggest that users shift blame away from themselves, we expect that the self-serving bias also occurs in interactions with service robots and hypothesize:

- H_2a_: If the service outcome is a failure (vs. success), the user attributes responsibility less internally.

#### Service recovery and responsibility attribution

Service research has long established that service failures are the main cause of user switching behavior (Keaveney, 1995) and the discontinued use of technologies (Sun et al., 2022). To retain users, firms need to transform the negative service outcome to a favorable one and assess which recovery strategy is most effective in interactions with service robots (Honig & Oron-Gilad, 2018; Reinkemeier & Gnewuch, 2022). Prior studies that have investigated different recovery strategies in interactions with service robots have thus far mainly focused on robot-initiated actions (Poser et al., 2021; Smith et al., 1999). Most commonly, research has examined the effectiveness of apologies and explanations (Choi et al., 2020; Honig & Oron-Gilad, 2018). Service literature recommends these two strategies because they effectively appease the user in the first step (Choi et al., 2020; Wei et al., 2020) and act as informative help (Colquitt, 2001). However, previous studies have shown that a recovery that provides immediate assistance received a better service evaluation than informative recovery strategies (Ho et al., 2020). Therefore, recently, researchers and firms are increasingly focusing on solving the problem through human handover (Choi et al., 2020; Honig & Oron-Gilad, 2018; Poser et al., 2021). This recovery strategy promises to be more effective in repairing damaged re-usage intentions because it can improve user experience through quick resolution (Kucherbaev et al., 2018) and can therefore mitigate dissatisfaction with the required service (Choi et al., 2020).

Prior work on service recovery has shown that recovery efforts are successful in attenuating negative user reactions after failure (e.g., Choi et al., 2020; Ho et al., 2020). However, it is unclear whether such recovery actions are seen by users as a firm’s admission of guilt (Gelbrich & Roschk, 2011) and hence reinforce their external blame attribution or whether such actions disrupt the “blame game”. Both internal and external responsibility attribution have the purpose of maintaining a positive self-image, so that users tend to attribute positive outcomes to themselves and negative outcomes to others (Campbell & Sedikides, 1999; Ross, 1977). External attribution can thus be understood in terms of a “self-protection strategy” (Campbell & Sedikides, 1999), which is particularly needed when the outcome is and remains negative (i.e., for service failures without recovery). Because of the immediate assistance through recovery, the failure is repaired and likely to be construed like a success. Users will therefore switch to attributional thinking similar to that of successful service delivery and are less likely to keep blaming external circumstances. In other words, through recovery, the need to self-protect should not be as prominent because the failure has been resolved and can be forgiven (Harrison-Walker, 2019). Hence, when firms provide recovery and thus change the service outcome to a favorable one, users should be more willing to take responsibility themselves so that internal attributions will be fostered (Dabholkar & Spaid, 2012). Therefore, we hypothesize:

- H_2b_: If the service failure is recovered (vs. not), the user attributes responsibility more internally.

### Social cognition theory

To evaluate how service robot design affects responsibility attribution in response to different service outcomes, we rely on social cognition theory. The following sections present prior work on service robot design and derive hypotheses for the effect of service robot design and the interaction effect of service outcome and service robot design on responsibility attribution.

#### Service robot design and responsibility attribution

Prior work suggests that user reactions to service robots depend on their design. More specifically, firms humanize service robots because this promises higher robot-level user retention (Blut et al., 2021). This happens because, with an increasingly human-like interface, users tend to perceive and treat robots as social beings (Nass & Moon, 2000; Nass et al., 1994). To conceptualize human-like service robot design, we rely on social cognition theory. This theory distinguishes between warmth and competence as two universal dimensions of social cognition (Fiske et al., 2007). Warmth describes whether the social counterpart intends something good and is often described with characteristics such as friendliness and trustworthiness (Cuddy et al., 2008; Fiske et al., 2007). Competence encompasses whether the social counterpart can accomplish certain purposes and is associated with intelligence and capability (Cuddy et al., 2008; Fiske et al., 2007). Prior research shows that these two dimensions of social cognition are also relevant in the case of humanoid service robots (Belanche et al., 2021; Choi et al., 2020). While warmth perceptions support relational expectations and outcomes, competence perceptions have a positive effect on transactional expectations and outcomes of the user-firm relationship (Belanche et al., 2021; Güntürkün et al., 2020). Overall, both warmth and competence perceptions seem to be positively related to subsequent user responses (Belanche et al., 2021; Wirtz et al., 2018). Notably, previous findings suggest that higher warmth perceptions are oftentimes associated with lower competence perceptions (Judd et al., 2005). Therefore, this research focuses on comparing the individual effects of warm vs. competent service robot design.

As stated above, high competence perceptions are associated with greater intelligence and ability. Therefore, users expect competent service robots to be capable of providing a desired outcome. As a consequence, the user will deem the robot more responsible for service outcomes and are less likely to internalize responsibility. However, for service robots with warm design, users do not ascribe them the capability to achieve an outcome to the same extent. Instead, the user should feel the robot has produced the service outcome only with the help of their efforts. Therefore, we suggest:

- H_3_: If service robots have a warm (vs. competent) design, the user attributes responsibility more internally.

#### The interplay of service failure, recovery, and service robot design

Limited prior work has investigated how the interplay of different service outcomes and service robot design affects user responses (Choi et al., 2020; Corti & Gillespie, 2016; Yam et al., 2021). As noted in H_2a_, we assume that service failures in interactions with service robots trigger the self-serving bias. In interactions between humans, attributional research has shown that the bias emerges between strangers. However, the bias is less pronounced or even non-existent if the persons involved are friends (Campbell et al., 2000). Warm robot design intends to elicit feelings of friendliness and relatedness (van Doorn et al., 2017) and is also associated with emotional values (Belanche et al., 2021), which typically occur in friendships. Moreover, Jörling et al. (2019) show that if people feel ownership over the service robot, negative outcomes are more likely to be internalized. The participants describe that they have established a relation to the service robot and thus perceive it as their responsibility to take care of the service robot and to assume responsibility. As previously pointed out, this type of relationship can occur especially with a warm service robot (Belanche et al., 2021; Güntürkün et al., 2020).

Therefore, we assume that a warm robot design will mitigate the negative (i.e., undesirable) effect of service failure on internal attribution responsibility and hypothesize the following:

- H_4a_: If a service failure occurs, internal responsibility attribution is higher for service robots with a warm (vs. competent) design.

Above, we propose that users can more easily build a personal connection to service robots with warm (vs. competent) design. Users seek to maintain this friend-like relationship by forgiving failures, especially if they are resolved and damage is prevented (van Doorn et al., 2017). This suggests that users should be more tolerant and sympathetic toward service robots with warm design (Fiske et al., 2007). As established in H_2b_, service recovery increases internal responsibility attribution for a failure compared to service failures without recovery, because users forgive the failure and are thus more likely to take part of the blame onto themselves. As prior work suggests bots with a human-like appearance are forgiven more easily (Corti & Gillespie, 2016; Yam et al., 2021), we assume that this effect will be reinforced after successful recovery. In other words, recovery efforts will be more likely to increase internal attribution for a service robot with a warm (vs. competent) design.

Hence, we hypothesize:

- H_4b_: If a service failure is recovered, internal responsibility attribution is higher for service robots with a warm (vs. competent) design.

## Empirical examination

To test our hypotheses, we conducted two empirical studies. Both studies apply a 3 (service outcome: success vs. failure vs. failure with recovery) ✕ 2 (service robot design: warm vs. competent) between-subject design, where participants are randomly assigned to one of the six experimental groups.

### Study 1

The goal of study 1 was to initially test our research propositions for robot-level responses as the baseline because the robot is the service agent delivering the service outcomes. Study 1 is set in a medical diagnosis context.

#### Design and sample

We recruited participants from a European university by using distribution lists and social media. The participants did not need to fulfill any prerequisites to take part in the study. After the survey, participants could take part in an optional raffle of online shopping vouchers. We chose a scenario-based approach to ensure that the interactions were identical except for the respective manipulations. In doing so, we could control for confounding influences to achieve high internal validity. After a brief introduction to the survey, we instructed participants to imagine they were feeling ill and seek medical assistance. When they arrived at the doctor's office, they were greeted by a humanoid service robot. Participants faced the humanoid service robot as a static image.

In both service robot design scenarios, participants saw a version of the service robot Cruzr by Ubtech Robotics (Ubtech, 2022). Both versions of the service robot design include a humanoid form, in that the robot has arms and a torso. For the warm service robot design, we chose to include features of a humanoid face because prior studies show that these human-like features foster perceptions of human warmth through purporting social capabilities (Choi et al., 2020; van Doorn et al., 2017). In contrast, the face of the competent service robot merely consisted of a display with no further human-like features. This was done to make the robot appear more machine-like and consequently less warm and more efficient, as competence perceptions are fostered through functional elements (Breazeal, 2003). Beyond the robots' appearances, we manipulated warmth and competence perceptions through their greetings at the beginning of the scripted interactions. After this introduction, we asked participants to rate their competence and warmth perceptions of the service robot. Next, we instructed the participants to imagine describing their symptoms to the service robot. In the success scenario, the robot would give the participant their diagnosis, while in the failure scenario, the robot stated that it is unable to give a diagnosis. In the recovery scenario, the service robot informed the participants that they will be transferred to a human physician, who would provide a diagnosis. We chose human handover as a recovery strategy because prior work suggests it is an effective means of mitigating negative user responses after failures (Poser et al., 2021). The diagnosis given in the recovery scenario was identical to the diagnosis in the service success scenario. For a full overview of the stimulus material, see Appendix 1.

After the experiment, participants answered manipulation checks on service outcomes. We then collected the measures on internal responsibility attribution and robot-level user retention. The survey closed with demographics and attention checks. Except for demographics, we measured all items on 7-point Likert scales anchored by 1 = strongly disagree to 7 = strongly agree, if not stated otherwise. For a full overview of all items, including reliability and validity measures, see Appendix 2.

The initial sample consisted of 349 participants. We discarded those who did not correctly recall and identify how the service robot looked like (23 participants) and those who self-reported that they did not fill out the survey conscientiously (1 participant) from further analyses. Therefore, the final sample comprises 325 participants (75% female, *M*_age_ = 30 years), which are randomly and approximately evenly distributed across the six scenarios. There was no significant difference between scenarios regarding participants’ prior experience with service robots and socio-demographics (all *p* > 0.1). Overall, the participants perceived the scenarios as realistic (*M* = 4.8, *SD* = 1.9, mean is significantly higher than scale midpoint: *t* = 7.63, *p* < 0.001).

#### Results

##### Manipulation checks

The manipulation checks for perceived service outcome in terms of success vs. failure (*M*_success_ = 4.36, *SD* = 1.68; *M*_failure_ = 1.55, *SD* = 1.23; *t* = 14.10, *p* < 0.001) and failure vs. failure with recovery (*M*_failure_ = 1.67, *SD* = 1.34; *M*_recovery_ = 2.06, *SD* = 1.74; *t* = –1.84, *p* < 0.05) were successful. Furthermore, the manipulation check for perceived competence shows that the service robot with competent design is perceived as significantly more competent than the service robot with warm design (*M*_competent_ = 4.54, *SD* = 1.32; *M*_warm_ = 4.16, *SD* = 1.40; *t* = 2.52, *p* < 0.01). Correspondingly, the manipulation check for perceived warmth shows that the service robot with a warm design is perceived as significantly warmer than the service robot with a competent design (*M*_competent_ = 3.81, *SD* = 1.34; *M*_warm_ = 4.31, *SD* = 1.35; *t* = –3.30, *p* < 0.001). This shows that the manipulation of service robot design was also successful. Interestingly, these results provide evidence for the fact that higher warmth perceptions are associated with lower competence perceptions and vice versa.

##### Main and interaction effects

**Fig. 2:**
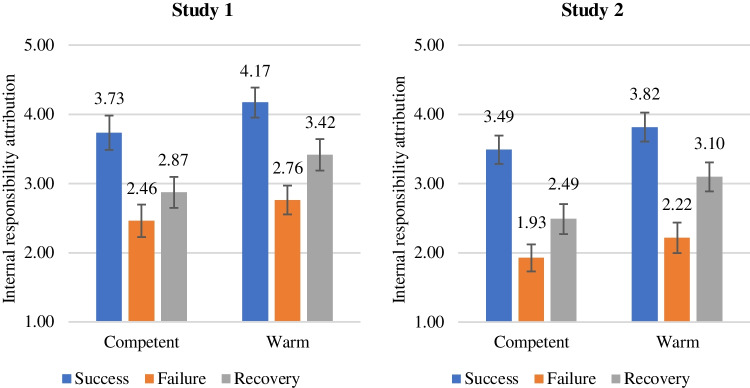
Interaction effect of service outcome and service robot design on internal responsibility attribution

To test the main effect postulated in H_1a_, we conducted regression analysis with robot-level user retention as the dependent variable and internal responsibility attribution as the independent variable. There is a significant positive effect of internal responsibility attribution on robot-level user retention (*β* = 0.19, *SE* = 0.05; *t* = 3.57, *p* < 0.001), which confirms our hypothesis. Furthermore, the effect remains significant if the direct main effects of service outcome, service robot design, and the interaction effect are included in the model. To test hypotheses H_2_, H_3,_ and H_4_, we conducted an analysis of variance (ANOVA) with internal responsibility attribution as the dependent variable and service outcome, service robot design as well as their interaction as independent variables. ANOVA results show a significant main effect of service outcome on internal responsibility attribution (*F*(2, 319) = 17.51, *p* < 0.001). Planned contrasts of predictive margins show that responsibility for service failure is attributed significantly less internally than for service success (*M*_success_ = 3.95, *SE* = 0.16; *M*_failure_ = 2.61, *SE* = 0.16; *t* = –5.89, *p* < 0.001), which provides support for H_2a_ and the existence of the self-serving bias in a service robot context. As assumed, if the service failure is recovered, the self-serving bias can be mitigated successfully and failure attribution shifts back internally (*M*_recovery_ = 3.14, *SE* = 0.16; *t* = 2.38, *p* < 0.05), which further provides support for H_2b_. Furthermore, ANOVA results show a significant main effect of service robot design on internal responsibility attribution (*F*(1, 319) = 5.30, *p* < 0.05). More precisely, outcomes are attributed significantly more internally for robots with warm than for robots with competent design (*M*_competent_ = 3.02, *SE* = 0.14; *M*_warm_ = 3.45, *SE* = 0.13; *t* = 2.3, *p* < 0.05), which provides support for H_3_. To analyze the interaction effect, we rely on planned contrasts. Figure 2 shows an overview of the interaction effect. Inconsistent with H_4a_, internal responsibility attribution is not higher for failures committed by a service robot with warm (vs. competent) design (*M*_competent✕failure_ = 2.46, *SE* = 0.23; *M*_warm✕failure_ = 2.76, *SE* = 0.21; *t* = 0.96, *p* > 0.1). However, warm (vs. competent) service robot design strengthens internal responsibility attribution for recovered failures (*M*_competent✕recovery_ = 2.87, *SE* = 0.22; *M*_warm✕recovery_ = 3.42, *SE* = 0.23; *t* = 1.7, *p* < 0.1), providing initial support for H_4b_.

##### Mediation analysis

To test for mediation, we use a bootstrapping procedure with 5000 iterations. Results reveal a negative indirect effect of failure (vs. success) (CI_95%_ = [–0.45, –0.11]), a positive indirect effect of recovery (vs. failure) (CI_95%_ = [0.02, 0.23]) and a positive indirect effect of warm (vs. competent) service robot design (CI_95%_ = [0.01, 0.18]) on robot-level user retention through internal responsibility attribution. Finally, while the indirect effect of the interaction of warm (vs. competent) service robot design and service failures is insignificant (CI_95%_ = [–0.05, 0.21]), the indirect effect of the interaction of warm (vs. competent) service robot design and service recovery through internal responsibility attribution on robot-level user retention is significant (CI_90%_ = [0.01, 0.25]).

### Study 2

To strengthen our findings, we conducted a second study. In addition to proving the robustness of first study’s effects on robot-level retention, we test whether the effects of the robot-delivered service outcomes and responsibility attribution spill over to firm-level user responses by including firm-level user retention. Furthermore, to elaborate on which entity the external responsibility attribution is shifted and specifically to test whether the blame for failure is shifted to the robot or the firm we conducted a post hoc analysis. Moreover, instead of a diagnosis service considered in study 1, we now examine a medical product recommendation.

#### Design and sample

For study 2, we recruited participants through the crowdsourcing platform Clickworker. Participants received monetary compensation and did not have to meet any specific requirements to participate on the study. We made minor adjustments to the service robot stimuli and the course of the interaction to fit the new context of study 2, which should also strengthen the robustness of our findings. Like study 1, study 2 is set in a medical context. However, instead of giving a medical diagnosis, the service robot gives a recommendation for medical treatment in study 2. Furthermore, while study 1 is set at a general practitioner's office, study 2 is set at a dermatologist's office. Apart from the minor changes to the stimulus material, the procedure of study 2 remains identical to that of study 1. All stimulus material is depicted in Appendix 3. In addition to the measures taken for study 1, we further included a second item to measure internal responsibility attribution, as well as a measurement of retention at the firm-level (i.e., the dermatologist's office) to test for H_1b_. As in study 1, we further included measures on demographics as well as a series of attention checks.

The initial sample consisted of 281 participants. We discarded those who failed attention checks (11 participants) and those who did not read the scenarios conscientiously, as they failed treatment checks (21 participants) from further analyses. Therefore, the final sample comprises 249 participants (42% female, *M*_age_ = 35 years), which are randomly and approximately evenly distributed across the six scenarios. Again, there was no significant difference between scenarios regarding participants’ prior experience with service robots and socio-demographics (all *p* > 0.1). Further, participants perceived the scenarios as realistic (*M* = 4.38, *SD* = 1.76, mean is significantly higher than scale midpoint: *t* = 3.45, *p* < 0.001).

#### Results

##### Manipulation checks

The results of the manipulation checks align with those of study 1. The manipulation checks for perceived service outcome in terms of success vs. failure (*M*_success_ = 5.45, *SD* = 1.17; *M*_failure_ = 1.21, *SD* = 0.58; *t* = 23.77, *p* < 0.001) and failure vs. failure with recovery (*M*_failure_ = 1.46, *SD* = 0.89; *M*_recovery_ = 5.94, *SD* = 1.22; *t* = –23.63, *p* < 0.001) were successful. As we adapted the design of the service robots compared to study 1, we tested the competence and warmth manipulation via a prestudy using a within-subject design (*N* = 40, 53% female, *M*_age_ = 27 years). The service robot with competent design is perceived as significantly more competent than the service robot with warm design (*M*_competent_ = 4.79, *SD* = 1.00; *M*_warm_ = 4.28, *SD* = 0.81; *t* = 3.05, *p* < 0.01). The service robot with warm design is perceived as significantly warmer than the service robot with competent design (*M*_competent_ = 3.7, *SD* = 1.07; *M*_warm_ = 4.62, *SD* = 1.06; *t* = –5.12, *p* < 0.001). Again, higher warmth perceptions are associated with lower competence perceptions and vice versa.

##### Main and interaction effects

To test the main effects of internal responsibility attribution on robot-level retention (H_1a_) and firm-level retention (H_1b_), we used regression analyses. There is a significant positive effect of internal responsibility attribution on robot-level user retention (*β* = 0.59, *SE* = 0.07; *t* = 8.10, *p* < 0.001), which confirms H_1a_. Additionally, we find a significant positive effect of internal responsibility attribution on firm-level user retention (*β* = 0.52, *SE* = 0.07; *t* = 7.04, *p* < 0.001), which provides support for H_1b_. Both effects remain significant if direct effects of service outcome, service robot design, and the interaction term are included in the models.

Results of ANOVA show a significant main effect of service outcome on internal responsibility attribution (*F*(2, 243) = 29.25, *p* < 0.001). Planned contrasts of predictive margins show that responsibility for service failure is significantly less internalized than for service success (*M*_success_ = 3.65, *SE* = 0.15; *M*_failure_ = 2.07, *SE* = 0.15; *t* = –7.64, *p* < 0.001), which provides support for H_2a_. If a service recovery occurs, internal responsibility increases compared to service failures without recovery (*M*_recovery_ = 2.79, *SE* = 0.15; *t* = 3.42, *p* < 0.001), providing support for H_2b_. Again, ANOVA results show a significant main effect of service robot design on internal responsibility attribution (*F*(1, 243) = 5.74, *p* < 0.05). In line with H_3_, internal responsibility attribution is higher for service robots with warm (vs. competent) design (*M*_competent_ = 2.63, *SE* = 0.12; *M*_warm_ = 3.04, *SE* = 0.12; *t* = 2.39, *p* < 0.05). We use planned contrasts to analyze the interaction effect, which is depicted in Fig. 2. In line with study 1, we find no support for H_4a_, as the internal responsibility attribution is not higher for failures committed by a service robots with warm (vs. competent) design (*M*_competent✕failure_ = 1.93, *SE* = 0.19; *M*_warm✕failure_ = 2.22, *SE* = 0.22; *t* = 0.98, *p* > 0.1). However, and in line with H_4b_, warm (vs. competent) service robot design strengthens internal responsibility attribution if service recovery occurs (*M*_competent✕recovery_ = 2.49, *SE* = 0.22; *M*_warm✕recovery_ = 3.10, *SE* = 0.21; *t* = 2.02, *p* < 0.05).

##### Mediation analysis

Mediation analysis using bootstrapping procedure reveals a negative indirect effect of failure (vs. success) on user retention (robot-level: CI_95%_ = [–1.25, –0.58]; firm-level: CI_95%_ = [–1.19, –0.55]), which is mediated by internal responsibility attribution. The indirect effect of recovery (vs. failure) through internal responsibility attribution on user retention is positive (robot-level: CI_95%_ = [0.18, 0.70]; firm-level: CI_95%_ = [0.14, 0.69]). Further, the warm (vs. competent) service robot design yields a positive indirect effect on user retention through internal responsibility attribution (robot-level: CI_95%_ = [0.05, 0.44]; firm-level: CI_95%_ = [0.05, 0.42]). In line with study 1, the indirect effect of the interaction of warm (vs. competent) service robot design and service failures on user retention is insignificant (robot-level: CI_95%_ = [–0.11, 0.46]; firm-level: CI_95%_ = [–0.11, 0.43]). Finally, there is an indirect effect of the interaction of warm (vs. competent) service robot design and service recovery on user retention (robot-level: CI_90%_ = [0.04, 0.71]; firm-level: CI_90%_ = [0.04, 0.66]).

In summary, both studies 1 and 2 confirm the majority of our hypotheses, except for H_4a_, which is rejected in both studies. Table 1 summarizes the results. Figure 2 visualizes the predictive margins of the interaction effect for both studies 1 and 2. For a complete overview of all ANOVAs for studies 1 and 2 results see Appendix 4.

**Table 1:** Overview of hypotheses

Hypothesis	Study 1	Study 2
H_1a_: Internal responsibility attribution increases robot-level user retention	✓	✓
H_1b_: Internal responsibility attribution increases firm-level user retention	n.a	✓
H_2a_: If the service outcome is a failure (vs. success), the user attributes responsibility less internally	✓	✓
H_2b_: If the service failure is recovered (vs. not), the user attributes responsibility more internally	✓	✓
H_3_: If service robots have a warm (vs. competent) design, the user attributes responsibility more internally	✓	✓
H_4a_: If a service failure occurs, internal responsibility attribution is higher for service robots with a warm (vs. competent) design	×	×
H_4b_: If a service failure is recovered, internal responsibility attribution is higher for service robots with a warm (vs. competent) design	✓^a^	✓

✓ = hypothesis supported, ×  = hypothesis rejected, n.a. = hypothesis not tested, ^a^ significant at 90%

#### Post hoc analysis

From the results of studies 1 and 2, it remains ambiguous to whom responsibility is attributed if it is not attributed internally. Therefore, to gain deeper insights into our results, we included more specific measures on external responsibility attribution in study 2, namely the robot's responsibility and the firm's responsibility. The items are included in Appendix 2. In specifying to whom or what responsibility is attributed in case of service failure, we enrich our studies' contributions.

For our post hoc analysis, we conducted two separate ANOVAs to test the individual effects of our treatments on the two dependent variables. Results show a significant main effect of service outcome on robot's responsibility (*F*(2, 243) = 3.02, *p* < 0.05) and firm's responsibility (*F*(2, 243) = 9.12, *p* < 0.001). Planned contrasts demonstrate that the difference between success and failure is insignificant in regards to the robot's responsibility (*M*_success_ = 4.63, *SE* = 0.19; *M*_failure_ = 5.03, *SE* = 0.19; *t* = 1.48, *p* > 0.1), however, there is a significant difference in regards to the firm's responsibility (*M*_success_ = 4.61, *SE* = 0.17; *M*_failure_ = 5.66, *SE* = 0.17; *t* = 4.25, *p* < 0.001). Interestingly, this suggests that the external attribution of responsibility observed in H_2a_ is directed at the firm, and not the robot. When it comes to the difference between failure and recovery, there is a significant difference in responsibility attribution toward the robot (*M*_failure_ = 5.03, *SE* = 0.19; *M*_recovery_ = 4.36, *SE* = 0.19; *t* = –2.44, *p* < 0.05) as well as toward the firm (*M*_failure_ = 5.66, *SE* = 0.17; *M*_recovery_ = 5.04, *SE* = 0.18; *t* = –2.47, *p* < 0.05). This suggests that both attribution of robot's responsibility and firm's responsibility decrease when internal responsibility attribution increases, as demonstrated in H_2b_.

**Fig. 3:**
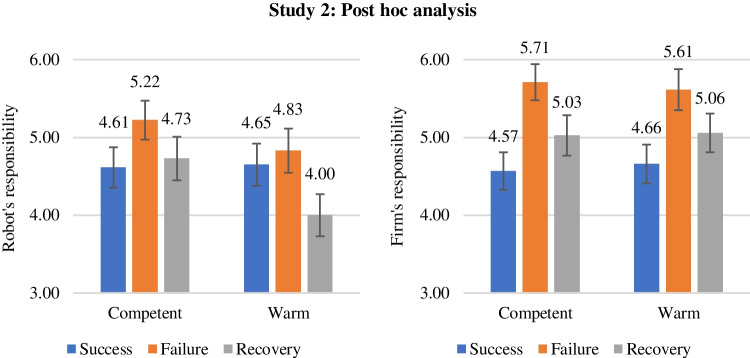
Interaction effect of service outcome and service robot design on external responsibility attribution

Finally, we find evidence for a significant difference in the attribution of the robot's responsibility after service recovery in the interaction with the warm (vs. competent) service robot (*M*_competent✕recovery_ = 4.73, *SE* = 0.28; *M*_warm✕recovery_ = 4.00, *SE* = 0.27; *t* = –1.87, *p* =  < 0.1). The same effect is insignificant with firm's responsibility as the dependent variable (*M*_competent✕recovery_ = 5.03, *SE* = 0.26; *M*_warm✕recovery_ = 5.06, *SE* = 0.25; *t* = 0.08, *p* > 0.1). This suggests that the increase in internal responsibility attribution after service recovery for warm (vs. competent) robots demonstrated in H_4b_ takes away blame from the warm robot, but not from the firm. The predictive margins of the interaction effect are depicted in Fig. 3. A complete overview of all post hoc analysis results can be found in Appendix 5.

## Discussion

The goal of the present work was to examine the extent to which users take responsibility for (i.e., internalize) different service outcomes and how this affects user retention. In this final chapter, we discuss our results and derive implications for theory and practice. Furthermore, we point out limitations of our research, which provide avenues for future research.

### Theoretical implications

Our research has several theoretical implications. First, our findings contribute to research on attributions of different service outcomes in a service robot context. Our studies highlight the positive relationship of internal responsibility attribution and robot- as well as firm-related user retention: If users internalize responsibility for an outcome, user retention increases. This happens because users like to feel in control of a situation, which is in line with findings from prior research on traditional service interactions (van Raaij & Pruyn, 1998). Our results further demonstrate that users claim responsibility for successes, but shift blame for failures away from themselves. The former implies that users believe they contributed to the successful outcome, in our case by communicating their health issue successfully. The latter however suggests that in case of a service failure, users deem external circumstances responsible. Thus, we contribute to attribution research in that our findings provide evidence for the self-serving bias in a service robot context and thus help dissipate prior inconsistent findings (Jörling et al., 2019; Leo & Huh, 2020).

Second, most of the previous service robot studies on attribution do not include behavioral outcomes that follow the attributional process. While a limited number of studies focuses on agent-level outcomes (e.g., Choi et al., 2020), there is barely any research that includes firm-level outcomes (Leo & Huh, 2020). Therefore, our studies addresses this research gap and investigate the impact of user retention for both agent and firm. Interestingly, our analysis suggests that the shift toward external responsibility attribution hits the firm, and not the robot, although the robot commits the failure. This finding suggests that robots are oftentimes seen as representatives of the firm, and not as individual actors (Belanche et al., 2020), therefore contributing to research on user reactions to a firm's agents vs. the firm itself (Palmatier et al., 2007). However, through a successful recovery, responsibility attribution “shifts back” toward internal attribution, away from the firm, which is predominantly in line with theory (Tsarenko & Strizhakova, 2013). Therefore, if a service failure is recovered, the negative effect of failure on user retention can be indirectly mitigated through increased internalization of responsibility.

Third, we contribute to existing research on robot service recovery by considering the impact of service recovery on the firm, as well as the consequences of human handover as a recovery strategy, rather than an apology or explanation, as it is often the case in research (e.g., Hu et al., 2021). Moreover, our research findings show the impact of the recovery strategy of human handover on attribution, which, to our knowledge, has not been examined in any other study so far. We can show that responsibility attribution is also a crucial mechanism in this context, explaining how users behave towards the service robot and firm.

Fourth, studies in the service failure context focus on the difference between a service robot and a human service agent (e.g., Ho et al., 2020; Leo & Huh, 2020; Merkle, 2019) but not how differently designed service robots affect, in particular, responsibility attribution. Our results contribute to social cognition theory in that they show that users attribute outcomes caused by service robots with warm design more internally, while they attribute outcomes caused by service robots with competent design more externally. On the one hand, this implies that users perceive robots with competent design as more capable and in charge of their actions, thus more responsible for outcomes, which is consistent with existing theory (Cuddy et al., 2008; Fiske et al., 2007). On the other hand, users perceive outcomes in interactions with robots with warm design as being created through the effort of the user. Such perceptions arise because users do not consider service robots with warm design as capable as service robots with competent design. Service robots with warm design lead to expectations of a trusting and friendly interaction rather than of competent and intelligent responses (Cuddy et al., 2008; Fiske et al., 2007).

Fifth, when taking a closer look at interactions of service outcome and service robot design, it becomes apparent that overall, the self-serving bias exists independently from social cognition in terms of robot design. The main effect of robot design described above is therefore outweighed by service failure, which is an interesting and valuable insight for research of robot design. Contrary to our hypothesis, internal responsibility attribution after service failure was not higher for robots with warm design. Attributional research suggests that the self-serving bias does not emerge in interactions between friends (Campbell et al., 2000). We assumed that through warm robot design, a similar result would be achieved. However, this was not the case, presumably because the brief interaction with the service robot was not able to elicit the same feelings of closeness that users feel in interactions with friends or ownership over the service robot. We further find that internal responsibility attribution after service recovery is higher if service robot design focuses on warmth. We assume this is because users are more forgiving toward a robot they perceive as friendly and good-natured (Lu et al., 2020). This finding is corroborated in our post hoc analysis which demonstrates that through recovery in response to failure committed by a service robot with a warm design, users are less likely to blame the robot and will take part of the responsibility onto themselves. Our studies thus make a novel contribution to current research on how robot design, in form of warmth and competence, can shape the impact of different service outcomes. Therefore, overall, our research provides valuable insights for the application of attribution theory and social cognition theory in a service robot context.

### Practical implications

Our findings also provide practical contributions. First, through lower internal responsibility attribution, service failures drastically diminish user retention. This effect is so severe that design considerations seem to become dispensable in failure situations. This suggests that firms need to increasingly invest efforts into further improving robot technology in order to minimize failures and thus customer churn. While we note that failures are inevitable in any service situation, it is still conceivable that further technological development allows for a noteworthy reduction of failure occurrences in HRI.

Second, our results suggest that once a failure occurs, users blame the firm and not the robot. Arguably, this happens because users see robots as an extension of the firm, and not as individual, sentient beings. This should be a warning to firms who see robots as individual agents instead of an extension of the firm. Firms need to consider carefully what kind of service robot they want to use (design, functionality, etc.) and ensure proactive recovery actions in case of a service failure.

Third, and interestingly, if failure is recovered, including features in robot design that elicit warmth perceptions helps firms retain users. Therefore, our results suggest that overall, service robots with warm design are suitable for all examined service outcomes, which maximizes the impact on user retention.

Fourth and finally, since our results are robust across both studies, the practical implications apply to different service settings. For example, in the first study the robot gave a medical diagnosis, and in the second study it recommended a medical product, so that we also provide practical insights for firm’s endeavors regarding the deployment of robo-advisors, which should also provide valuable insights for future applications of HRI, such as conversational commerce (Brengman et al., 2021; Lim et al., 2022; Schwede et al., 2022).

In sum, our research demonstrates that while firms should continue striving for failure-free service interactions with robots, they should focus on deploying warm robots as they are particularly effective when failures occur that can be recovered while performing equal to competent robots for successful service outcomes or non-recoverable service failures.

### Limitations and future research

The present study is not free of limitations, which open up avenues for further research. When considering attributional processes in response to different service outcomes, our work focuses on internal responsibility attribution. Traditionally, in attribution theory three types of attributions are investigated: locus of causality, controllability, and stability (Weiner, 2000). Extant studies have combined the former two to the dimension of responsibility attribution (Belanche et al., 2020), which is the variable examined here. Furthermore, we did not include stability perceptions in our framework, as prior work has found that stability attributions are of less relevance in failure situations (Leo & Huh, 2020; van Vaerenbergh et al., 2014). For a more nuanced overview of how user attributions explain the effect of different service failures and robot designs on usage intentions, future studies should consider including all dimensions as explanatory mechanisms.

Next, we considered warmth and competence dimensions for service robot design. The manipulation checks on perceived competence and warmth suggest that the two dimensions are mutually exclusive, at least as a result of our operationalization. However, in prior work, traits that stimulate warmth perceptions did not interfere with competence perceptions (Choi et al., 2020). Existing research has discussed under which circumstances the dimensions co-occur or not (Judd et al., 2005), however, this question remains unanswered in service robot research. Regarding the setting of our study, prior literature suggests that both warmth and competence perceptions are relevant for robot acceptance in a medical context (Laranjo et al., 2018). Future studies should consider manipulating different configurations and combinations of warmth and competence.

Moreover, the physical appearance of robots has been demonstrated to be vital to the perception and evaluation of robots (Blut et al., 2021). However, service robot design could not buffer the negative effects of failure in neither of our studies. Therefore, future research should further investigate different aspects of human-like service robot design in interactions with different service outcomes. Altogether, it becomes apparent that the effect of service failure mostly outweighs design considerations. For firms, this implies that investing effort in recovery strategies is of great relevance. In our study, it has been shown that human intervention mitigates consequences, especially for a robot with a warm design. Further studies could explore additional recovery strategies, such as recovery through the respective (or even another) robot. In addition, user characteristics (e.g., a priori user attitudes towards robots in general or ethical positions; Lim & Weissmann, 2021) and context characteristics (e.g., product vs. service; Lovelock & Wirtz, 2011; Pan & Siemens, 2011; or high- vs. low-status settings; Mattar, 2021) may also have an impact on the effect of service outcomes and service robot design. Further research should therefore include further personality characteristics in form of a before/after comparison as well as characteristics of behavioral control and situational factors that lead to behavioral changes.

Finally, our studies relied on descriptive scenarios with pictures of service robots, instead of a real service interaction. We did this to be able to control for confounding influences and to assure that the interactions are identical except for the respective manipulations. As a consequence, external validity may have been hampered (Aguinis & Bradley, 2014). To address this, future studies should examine real-life interactions between users and service robots.

## Supplementary Information

Below is the link to the electronic supplementary material.

Supplementary file1 (PDF 316 kb)
