# Supplementary material for: Users taking the blame? How service failure, recovery, and robot design affect user attributions and retention
Source: Electron Mark. 2023 Jan 19;32(4):2491–505. doi: 10.1007/s12525-022-00613-4 (PMC9849113; doi:10.1007/s12525-022-00613-4)
Supplement: Supplementary file 1 — Supplementary file1 (PDF 316 kb) [file 12525_2022_613_MOESM1_ESM.pdf]

Electronic Markets
The International Journal on Networked Business

Appendix

	Competence	Warmth
**Introduction**	Imagine that you feel like you have a cold and want to visit a doctor for this reason.<br>When you arrive at the doctor's office, a robot welcomes you.	
**Manipulation of service robot design**	The robot greets you with the following sentences:<br>"Hello, I am the humanoid Health Assistant X.<br>Thanks to the use of the latest technology, I can be of great help to you. Please tell me about your symptoms."<br>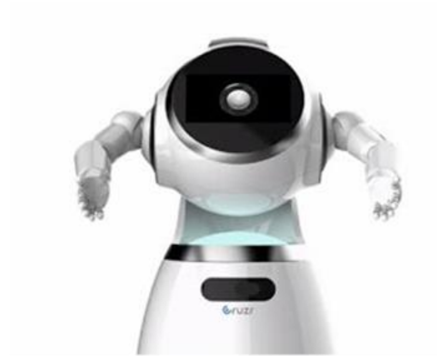	The robot greets you with the following sentences:<br>"Hello, I am DocRobot, nice to see you."<br>I am happy to help you. Please tell me what ails you."<br>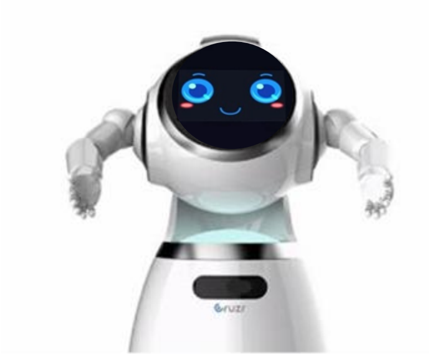
**Success**	Imagine that you describe your symptoms.<br>After a short moment, the Health Assistant X / DocRobot tells you that your symptoms point to a flu-like infection and that you should drink plenty of fluids and avoid demanding activities	
**Failure**	Imagine that you describe your symptoms.<br>After a short moment, the Health Assistant X / DocRobot tells you that it cannot understand your information and cannot assess what is wrong with you at the moment.	
**Failure with recovery**	Imagine that you describe your symptoms.<br>After a short moment, the Health Assistant X / DocRobot tells you that it cannot understand your information and cannot assess what is wrong with you at the moment.<br>After a short moment, the Health Assistant X / DocRobot asks you to come into the doctor's office. There, the doctor tells you that your symptoms point to a flu-like infection and that you should drink plenty of fluids and avoid demanding activities.	

Appendix 1: Stimulus material – Study 1

Constructs	Items	Item loadings
**Perceived warmth**<br>α = 0.890 (0.808)<br>adapted from Fiske et al., 2002; Güntürkün et al., 2020	The service robot is warm.	0.845 (0.729)
	The service robot is sincere.	0.895 (0.808)
	The service robot is friendly.	0.875 (0.842)
	The service robot is trustworthy.	0.858 (0.814)
**Perceived competence**<br>α = 0.908 (0.867)<br>adapted from Fiske et al., 2002; Güntürkün et al., 2020	The service robot is competent.	0.925 (0.911)
	The service robot is capable.	0.928 (0.915)
	The service robot is intelligent.	0.899 (0.789)
	The service robot is efficient.	0.792 (0.776)
**Service outcome (Success vs. failure)**<br>adapted from Lastner et al., 2016	The service robot provided a satisfactory recommendation for my inquiry. (The robot could solve my service inquiry.)	single item
**Service outcome (Recovery vs. no recovery)**<br>adapted from Lastner et al., 2016; Maxham & Netemeyer, 2002	The dermatologist's office provided a satisfactory recommendation for my inquiry. (A solution was provided to my problem.)	single item
**Internal responsibility attribution**<br>α = 0.828 (n.a.)<br>adapted from Kim & Smith, 2005	The outcome of the service interaction: (7) lies inside of my responsibility / (1) lies outside of my responsibility	0.925 (n.a.)
	The outcome of the service interaction: (7) happened because of me / (1) happened because of someone else[a]	0.925 (n.a.)
**Robot-level user retention**<br>α = 0.954 (0.951)<br>adapted from Venkatesh & Davis, 2000; Heerink et al., 2010	If I had the choice, I would intend to use the service robot again.	0.964 (0.936)
	If I had the choice, I would probably not use the service robot again.[r] (If given the chance, I intend to use the service robot in the near future)	0.955 (0.971)
	If I had the choice, I would use the service robot again next time. (If given the chance, I think I'll use the service robot in the near future)	0.955 (0.958)
**Firm-level user retention**[a]<br>α = 0.953 (n.a.)<br>adapted from Palmatier et al., 2007	For my next dermatologist appointment, I would consider this dermatologist's office as my first choice.[a]	0.955 (n.a.)
	I would come back to this dermatologist's office in the future.[a]	0.960 (n.a.)
	All else being equal, I would continue to visit this dermatologist's office in the future.[a]	0.954 (n.a.)
**Robot's responsibility**[a]<br>α = 0.941 (n.a.)<br>adapted from Lei & Rau, 2021	The service outcome was primarily dependent on (7) the service robot / (1) something else. [a]	0.933 (n.a.)
	The responsibility for the service outcome lies primarily with (7) the service robot / (1) something else. [a]	0.949 (n.a.)
	The responsibility for the service outcome predominantly depends on (7) the service robot / (1) something else. [a]	0.955 (n.a.)
**Firm's responsibility**[a]<br>α = 0.932 (n.a.)<br>adapted from Lei & Rau, 2021	The service outcome was primarily dependent on (7) the dermatologist's office / (1) something else. [a]	0.901 (n.a.)
	The responsibility for the service outcome lies primarily with (7) the dermatologist's office / (1) something else. [a]	0.963 (n.a.)
	The responsibility for the service outcome predominantly depends on (7) dermatologist's office / (1) something else. [a]	0.952 (n.a.)
**Scenario realism**<br>adapted from Bagozzi et al., 2016	The presented scenario was realistic.	single item

Notes: α = Cronbach's alpha; n.a. = not applicable; [a] not collected in study 1; [r] reverse scaled; text and values in parentheses refer to study 1

Appendix 2: Measurements – Study 1 and 2

	Competence	Warmth
**Introduction**	Imagine you have discovered a reddish rash on your arm. You conclude that the rash is caused by your new detergent. However, the rash is itchy and painful and you want to visit a dermatology office to get a recommendation for a suitable ointment.<br><br>When you arrive at the dermatologist's office, a robot welcomes you.	
**Manipulation of service robot design**	The robot greets you with the following sentences:<br><br>"Hello, I am the intelligent service robot XR3000.<br><br>Thanks to the use of the latest AI technology, I can advise you efficiently and competently."<br><br>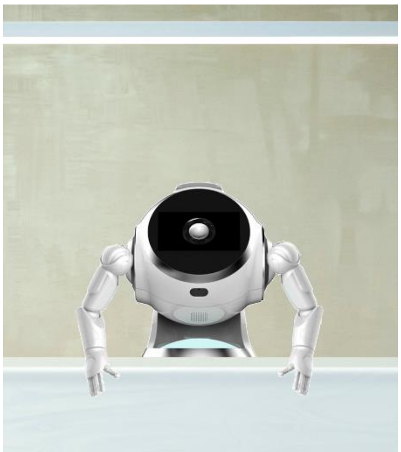	The robot greets you with the following sentences:<br><br>"Hey, I am your friendly service robot named Kim.<br><br>I am very happy to help you."<br><br>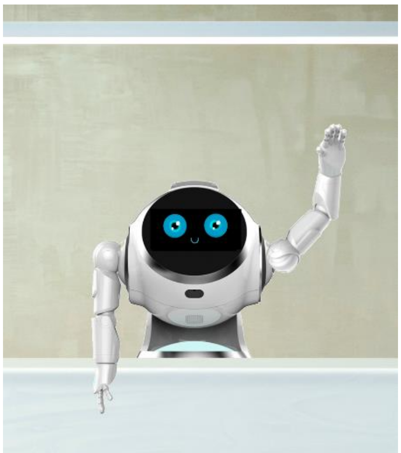
**Success**	Imagine that you describe your symptoms and the cause for your rash to the robot. Then you ask it for a recommendation for a suitable ointment.<br><br>After a short moment, the service robot XR3000 / Kim recommends the ointment Cepanthen Sensiderm. You should apply it to the red and itchy skin. The ointment is suitable for stabilizing the skin's protective barrier in case of skin irritations.	
**Failure**	Imagine that you describe your symptoms and the cause for your rash to the robot. Then you ask it for a recommendation for a suitable ointment.<br><br>After a short moment, the service robot XR3000 / Kim tells you that it cannot give you a recommendation at the moment.	
**Failure with recovery**	Imagine that you describe your symptoms and the cause for your rash to the robot. Then you ask it for a recommendation for a suitable ointment.<br><br>After a short moment, the service robot XR3000 / Kim tells you that it cannot give you a recommendation at the moment.<br><br>After a short moment, the service robot XR3000 / Kim asks you to come into the dermatologist's office. There, the dermatologist recommends the ointment Cepanthen Sensiderm. You should apply it to the red and itchy skin. The ointment is suitable for stabilizing the skin's protective barrier in case of skin irritations.	

Appendix 3: Stimulus material – Study 2

	**Dependent variable: Internal responsibility attribution**			
	**Study 1**		**Study 2**	
**Main effect:**<br>Service outcome	$F(2,319) = 17.51$, $p < 0.001$		$F(2,243) = 29.25$, $p < 0.001$	
	$M_{\text{Success}} = 3.95$,<br>$SE = 0.16$<br><br>$M_{Failure} = 2.61$,<br>$SE = 0.16$<br><br>$t = -5.89$, $p < 0.001$	$M_{failure} = 2.61$,<br>$SE = 0.16$<br><br>$M_{recovery} = 3.14$,<br>$SE = 0.16$<br><br>$t = 2.38$, $p < 0.05$	$M_{\text{success}} = 3.65$,<br>$SE = 0.15$<br><br>$M_{failure} = 2.07$,<br>$SE = 0.15$<br><br>$t = -7.64$, $p < 0.001$	$M_{failure} = 2.07$,<br>$SE = 0.15$<br><br>$M_{recovery} = 2.79$,<br>$SE = 0.15$<br><br>$t = 3.42$, $p < 0.001$
**Main effect:**<br>Service robot design	$F(1,319) = 5.30$, $p < 0.05$		$F(1,243) = 5.74$, $p < 0.05$	
	$M_{competent} = 3.02$, $SE = 0.14$<br><br>$M_{warm} = 3.45$, $SE = 0.13$<br><br>$t = 2.30$, $p < 0.05$		$M_{competent} = 2.63$, $SE = 0.12$<br><br>$M_{warm} = 3.04$, $SE = 0.12$<br><br>$t = 2.39$, $p < 0.05$	
**Interaction effect:**<br>Service outcome × Service robot design	$F(2, 319) = 0.14$, $p = 0.866$		$F(2, 243) = 0.34$, $p = 0.71$	
	$M_{competentxfailure} = 2.46$,<br>$SE = 0.23$<br><br>$M_{warmxfailure} = 2.76$,<br>$SE = 0.21$<br><br>$t = 0.96$, $p = 0.338$	$M_{competentxrecovery} = 2.87$, $SE = 0.22$<br><br>$M_{warmxrecovery} = 3.42$,<br>$SE = 0.23$<br><br>$t = 1.70$, $p = 0.091$	$M_{competentxfailure} = 1.93$,<br>$SE = 0.19$<br><br>$M_{warmxfailure} = 2.22$,<br>$SE = 0.22$<br><br>$t = 0.98$, $p = 0.326$	$M_{competentxrecovery} = 2.49$, $SE = 0.22$<br><br>$M_{warmxrecovery} = 3.10$,<br>$SE = 0.21$<br><br>$t = 2.02$, $p < 0.05$

Appendix 4: Overview of ANOVA results – Study 1 and 2

	Dependent variable			
	**Robot‘s responsibility**		**Firm‘s responsibility**	
**Main effect:** Service outcome	$F(2, 243) = 3.02, p < 0.05$		$F(2, 243) = 9.12, p < 0.001$	
	$M_{\text{Success}} = 4.63$, $SE = 0.19$	$M_{failure} = 5.03$, $SE = 0.19$	$M_{\text{success}} = 4.61$, $SE = 0.17$	$M_{failure} = 5.66$, $SE = 0.17$
	$M_{Failure} = 5.03$, $SE = 0.19$	$M_{recovery} = 4.36$, $SE = 0.19$	$M_{failure} = 5.66$, $SE = 0.17$	$M_{recovery} = 5.04$, $SE = 0.18$
	$t = 1.48, p = 0.141$	$t = -2.44, p < 0.05$	$t = 4.25, p < 0.001$	$t = -2.47, p < 0.05$
**Main effect:** Service robot design	$F(1, 243) = 2.71, p = 0.101$		$F(1, 243) = 0.00, p = 0.97$	
	$M_{competent} = 4.85, SE = 0.15$		$M_{competent} = 5.10, SE = 0.14$	
	$M_{warm} = 4.49, SE = 0.16$		$M_{warm} = 5.11, SE = 0.15$	
	$t = -1.65, p = 0.101$		$t = 0.04, p = 0.967$	
**Interaction effect:** Service outcome × Service robot design	$F(2, 243) = 1.01, p = 0.365$		$F(2, 243) = 0.07, p = 0.929$	
	$M_{competentxfailure} = 5.22$, $SE = 0.25$	$M_{competentxrecovery} = 4.73$, $SE = 0.28$	$M_{competentxfailure} = 5.71$, $SE = 0.23$	$M_{competentxrecovery} = 5.03$, $SE = 0.26$
	$M_{warmxfailure} = 4.83$, $SE = 0.28$	$M_{warmxrecovery} = 4.00$, $SE = 0.27$	$M_{warmxfailure} = 5.61$, $SE = 0.26$	$M_{warmxrecovery} = 5.06$, $SE = 0.25$
	$t = -1.04, p = 0.299$	$t = -1.87, p = 0.062$	$t = -0.27, p = 0.785$	$t = 0.08, p = 0.932$

Appendix 5: Overview of ANOVA results for post hoc analysis – Study 2

References

Bagozzi, R. P., Belanche, D., Casaló, L. V., & Flavián, C. (2016). The Role of Anticipated Emotions in Purchase Intentions. *Psychology & Marketing*, *33*(8), 629–645. https://doi.org/10.1002/mar.20905

Fiske, S. T., Cuddy, A. J. C., Glick, P., & Xu, J. (2002). A model of (often mixed) stereotype content: Competence and warmth respectively follow from perceived status and competition. *Journal of Personality and Social Psychology*, *82*(6), 878–902. https://doi.org/10.1037//0022-3514.82.6.878

Güntürkün, P., Haumann, T., & Mikolon, S. (2020). Disentangling the Differential Roles of Warmth and Competence Judgments in Customer-Service Provider Relationships. *Journal of Service Research*, *23*(4), 476–503. https://doi.org/10.1177/1094670520920354

Heerink, M., Kröse, B., Evers, V., & Wielinga, B. (2010). Assessing Acceptance of Assistive Social Agent Technology by Older Adults: the Almere Model. *International Journal of Social Robotics*, *2*(4), 361–375. https://doi.org/10.1007/s12369-010-0068-5

Kim, Y. S. K., & Smith, A. K. (2005). Crime and Punishment. *Journal of Service Research*, *8*(2), 162–180. https://doi.org/10.1177/1094670505279418

Lastner, M. M., Folse, J. A. G., Mangus, S. M., & Fennell, P. (2016). The road to recovery: Overcoming service failures through positive emotions. *Journal of Business Research*, *69*(10), 4278–4286. https://doi.org/10.1016/j.jbusres.2016.04.002

Lei, X., & Rau, P. L. P. (2021). Effect of relative status on responsibility attributions in human–robot collaboration: Mediating role of sense of responsibility and moderating role of power distance orientation. *Computers in Human Behavior*, *122*, 106820. https://doi.org/10.1016/j.chb.2021.106820

Maxham, J. G., & Netemeyer, R. G. (2002). A Longitudinal Study of Complaining Customers' Evaluations of Multiple Service Failures and Recovery Efforts. *Journal of Marketing*, *66*(4), 57–71. https://doi.org/10.1509/jmkg.66.4.57.18512

Palmatier, R. W., Scheer, L. K., & Steenkamp, J. B. E. (2007). Customer Loyalty to Whom? Managing the Benefits and Risks of Salesperson-Owned Loyalty. *Journal of Marketing Research*, *44*(2), 185–199. https://doi.org/10.1509/jmkr.44.2.185

Venkatesh, V., & Davis, F. D. (2000). A Theoretical Extension of the Technology Acceptance Model: Four Longitudinal Field Studies. *Management Science*, *46*(2), 186–204. https://doi.org/10.1287/mnsc.46.2.186.11926
